# Supplementary material for: Splice-Junction-Based Mapping of Alternative Isoforms in the Human Proteome
Source: Cell Rep. Author manuscript; Available in PMC 2020 Jan 15. (PMC6961840; doi:10.1016/j.celrep.2019.11.026)
Supplement: 2 [file NIHMS1546469-supplement-2.zip › DF1/PXD009737/TestisLysC/TestisLysC_2_DLG1_NEPDRALHLLEEYRSK.pdf]

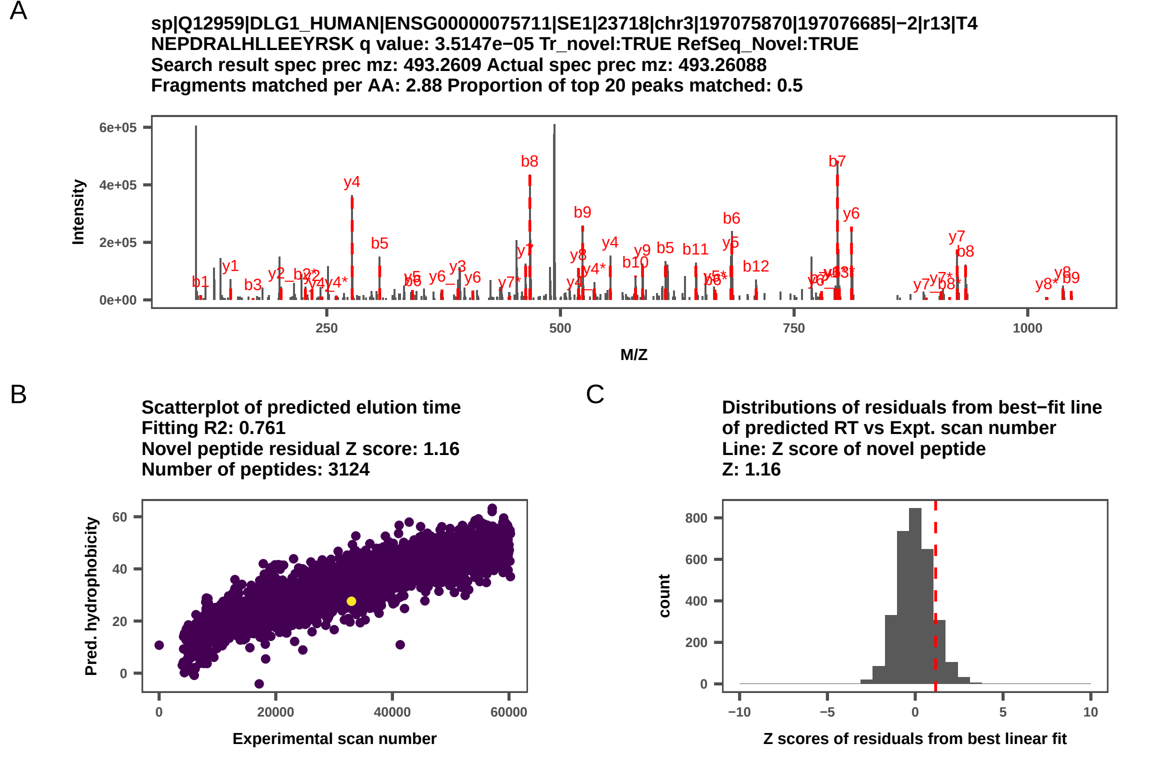
A
sp|Q12959|DLG1_HUMAN|ENSG00000075711|SE1|23718|chr3|197075870|197076685|−2|r13|T4
NEPDRALHLLEEYRSK q value: 3.5147e−05 Tr_novel:TRUE RefSeq_Novel:TRUE
Search result spec prec mz: 493.2609 Actual spec prec mz: 493.26088
Fragments matched per AA: 2.88 Proportion of top 20 peaks matched: 0.5
Intensity
6e+05
4e+05
2e+05
0e+00
M/Z
250
500
750
1000
y4
b8
b7
y6
b9
b6
y7
b8
b5
y1
b1
b3
y4
b10
y9
b5
b11
b12
B
Scatterplot of predicted elution time
Fitting R2: 0.761
Novel peptide residual Z score: 1.16
Number of peptides: 3124
Pred. hydrophobicity
Experimental scan number
0
20
40
60
0
20000
40000
60000
C
Distributions of residuals from best−fit line
of predicted RT vs Expt. scan number
Line: Z score of novel peptide
Z: 1.16
count
Z scores of residuals from best linear fit
0
200
400
600
800
−10
−5
0
5
10
